# Uncertainty quantification in FEM simulation of human liver: sensitivity analysis, Gradient-Enhanced Kriging, and Monte Carlo simulation

**DOI:** 10.1007/s10237-026-02084-4

**Published:** 2026-07-30

**Authors:** Navina Waschinsky, Carmen van Meegen, Lena Lambers, Katja Ickstadt, Tim Ricken

**Affiliations:** 1 Faculty of Aerospace Engineering and Geodesy, Institute of Structural Mechanics and Dynamics in Aerospace Engineering (ISD), University of Stuttgart, Pfaffenwaldring 27, 70191 Stuttgart, Germany; 2 Chair of Mathematical Statistics and Biometric Applications, Department of Statistics, TU Dortmund University, Vogelpothsweg 87, 44221 Dortmund, Germany

**Keywords:** Uncertainty quantification, Surrogate modeling, Sensitivity analysis, Multiscale and multiphase modeling, Computational liver model

## Abstract

The numerical simulation of metabolic processes in the human liver involves challenges related to model accuracy, physiological representation, and data uncertainty. Soft tissue models must capture complex, multiphase, and time-dependent behavior. However, the high computational cost of such analyses often restricts them to simplified deterministic scenarios that neglect biological variability and uncertainty. In this paper, the deterministic foundation of a liver perfusion-function model based on the Finite Element Method (FEM) is extended to incorporate material variability. To this end, a workflow is presented that integrates sensitivity analysis, surrogate modeling based on a Gradient-Enhanced Kriging (GEK) model, and uncertainty quantification. This approach enables the identification of critical parameters, enhances the interpretability of the model, and provides probabilistic outcome assessments for FEM-based simulations. The FEM model represents liver tissue on the lobule scale, incorporating metabolically active cells and vascular systems through a poroelastic multiscale framework with spatially coupled function-perfusion dynamics. It simulates liver-related conditions, such as tumor growth due to metabolic associated fatty liver disease (MAFLD), which affects microperfusion. Aleatoric uncertainties in material parameters are accounted for, and a local sensitivity analysis identifies critical nodes as a foundation for a GEK metamodel. Monte Carlo (MC) methods on the surrogate enable fast propagation of input uncertainty and probabilistic assessment of predicted outcomes. Rather than assuming deterministic accuracy, this approach offers a systematic way to explore and interpret model behavior under uncertainty.

**Supplementary Information:**

The online version contains supplementary material available at https://doi.org/10.1007/s10237-026-02084-4.

## Introduction

Increasing the fidelity of simulations, defined as the extent to which a computational model accurately reflects the underlying physical and biochemical processes, enhances realism but also significantly increases model complexity and computational cost. Simulating the human liver presents significant challenges due to its anatomical and physiological complexity. Key challenges include multiscale dynamics, ranging from organ-level behaviors to microscale interactions, the heterogeneity of liver tissue, fluid–structure interactions, and the complex biochemical processes essential for metabolism. This complexity requires sophisticated modeling approaches to accurately reproduce liver function and predict physiological responses under different conditions. A previously developed scale-bridging and multiphysical numerical model of the function-perfusion-structure interaction of the liver, see Ricken et al. (2010), Ricken et al. (2015), Ricken et al. (2018), Ricken and Lambers (2019), and Lambers et al. (2024), addresses some of these complexities. This model integrates the metabolically active cellular level with vascular systems using the extended Theory of Porous Media (eTPM). The eTPM is a macroscopically homogenized continuum mechanical model approach that was originally developed in engineering, see Bluhm et al. (2011), Moj et al. (2017), Thom and Ricken (2019), and Seyedpour et al. (2023b). It offers an excellent opportunity to accurately and computational efficiently map deformation, flow and transport processes in porous media, cf. Seyedpour et al. (2019). The mathematical model simulates spatially and temporally varying blood perfusion at the liver lobule scale, coupled with metabolic activity at the microscale. Applying transverse isotropic permeability simulates hepatic blood flow and metabolic functions like protein, carbohydrate, and lipid metabolism along the port-central axis. While this model marks a significant step forward, it also introduces more uncertainties. The increasing number of parameters and interactions complicates the model’s validation and calibration with experimental data. Moreover, the complexity of the model raises computational demands and the likelihood of errors due to potential inaccuracies in parameter estimation and to the natural variability of biological systems. In addition to macroscopic scale-bridging models, scale-separating multiscale approaches with computational homogenization, see Ricken et al. (2022), as well as non-scale-separating methods, see Maike et al. (2024), are also available. These methods allow for a more accurate resolution of the microstructure, including the interactions that occur at the microscale. However, this requires significantly higher computational effort. Since the investigation of uncertainty quantification itself already demands high computational resources, computational homogenization methods will not be discussed further in this paper.

Addressing sensitivity and uncertainty in the numerical liver model is essential to ensure transparent predictions and to assess model credibility. These uncertainties arise from the variability of biological parameters, experimental inaccuracies, limitations of accurate in vivo experiments, limited data quality, and poorly defined boundary conditions. Verification, validation, and uncertainty quantification form the methodological foundation for assessing the trustworthiness of numerical results (Hauseux et al. 2018). Sensitivity analysis has proven instrumental in identifying critical parameters influencing liver simulations, as demonstrated by Suwelack et al. (2011). Furthermore, uncertainty quantification techniques such as the Unscented Kalman Filter have facilitated the integration of patient-specific boundary conditions, allowing for variability in anatomical and biomechanical properties to be considered (Nikolaev and Cotin 2020). Within the general framework of the Theory of Porous Media (TPM), uncertainty quantification has been investigated for engineering applications (Schmidt et al. 2019; Seyedpour et al. 2023a). In contrast, the present work applies uncertainty quantification to the eTPM formulation for liver tissue.

Due to the high computational cost of complex liver simulations, surrogate models or emulators are essential to enable uncertainty analyses in a computationally efficient manner. Surrogate modeling techniques, such as Gaussian process models, provide a pathway for efficient uncertainty quantification in complex numerical models, see Lee et al. (2020). Furthermore, Pellicer-Valero et al. (2020) propose a machine learning model trained on a large dataset of finite element simulations of liver tissue, enabling real-time biomechanical predictions across various liver geometries and material properties. This approach demonstrates how data-driven surrogate models can efficiently capture complex biomechanical behavior while addressing uncertainty and variability.

The aim of this paper is to overcome the limitations of deterministic liver simulations by integrating uncertainty quantification. Building upon the FEM-based eTPM model of Ricken et al. (2010, 2018) and Lambers et al. (2024), we enable efficient, probabilistic predictions of perfusion–function dynamics and MAFLD-driven tumor growth. The approach combines structural analysis using the eTPM framework with local sensitivity analysis, Latin Hypercube Sampling (LHS), GEK surrogates, and MC simulations to propagate input variability and provide a comprehensive assessment of liver behavior under realistic physiological conditions. Specifically, the objectives of this paper are as follows:

1. Identify critical parameters through local sensitivity analysis.
2. Employ a GEK metamodel for efficient uncertainty propagation.
3. Systematically evaluate the uncertainty in the material parameters.
4. Uncertainty distribution for the results of the numerical simulation assessing the impact of hepatocellular carcinoma (HCC) in a liver lobule.

Sect. 2.1 introduces the general workflow. The workflow includes the FEM model in Sect. 2.2, the local sensitivity analysis in Sects. 2.3 as well as the GEK model described in Sect. 2.4. Results and discussions are presented in Sect. 3, concluding with a discussion in Sect. 4.

## Workflow and methods

### Workflow

**Fig. 1:**
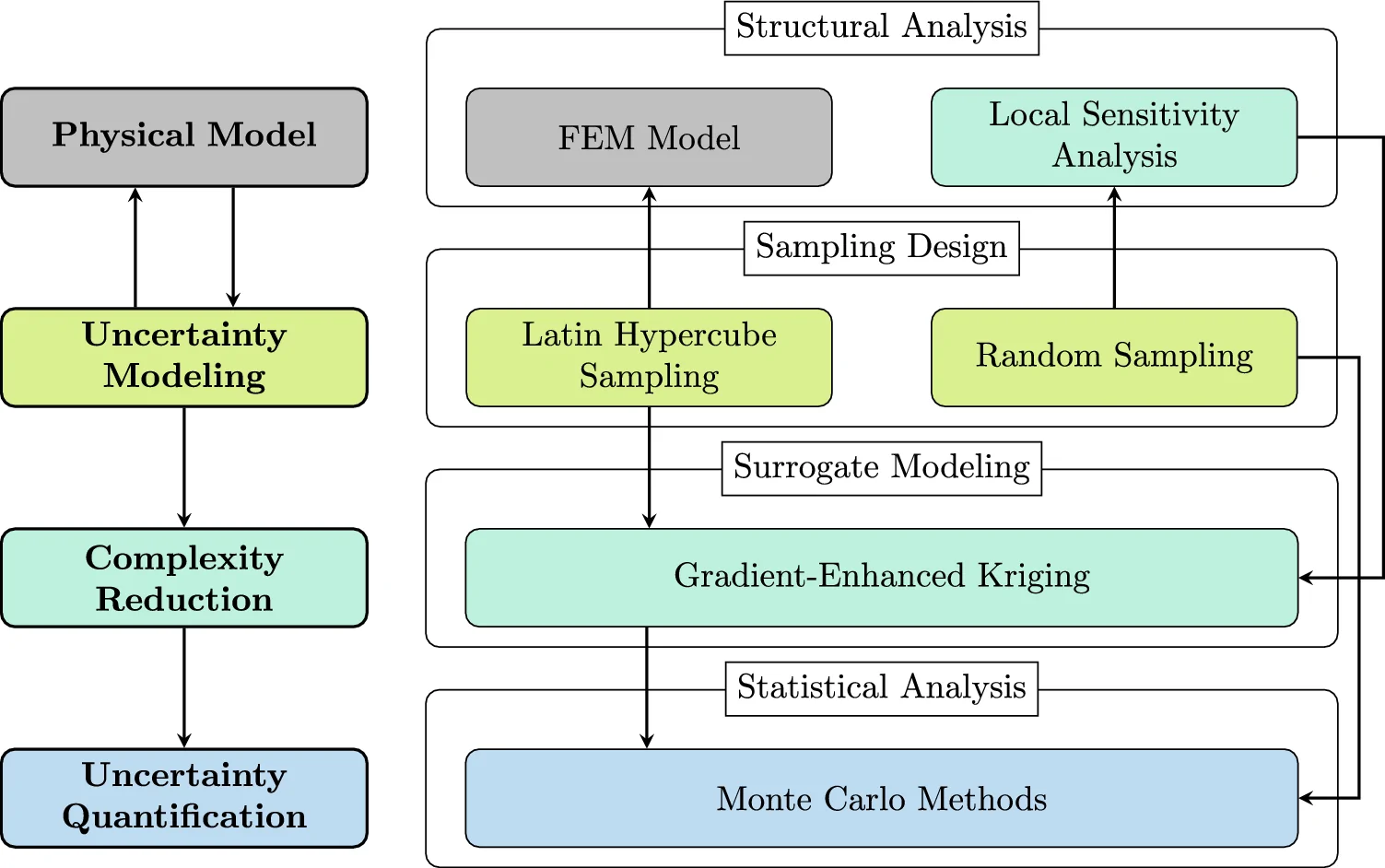
The workflow consists of four stages: 1. Physical Model, 2. Uncertainty Modeling, 3. Complexity Reduction and 4. Uncertainty Quantification. The interconnection between these components plays a crucial role in effectively quantifying and propagating uncertainties in complex structural simulations

Figure 1 outlines a workflow for FEM-based sensitivity analysis and uncertainty quantification, based on the results of the Priority Program (SPP) 1886, SP12, van Meegen et al. (2025). The left-hand side of the figure outlines the sequential steps of the workflow. These are thematically grouped into four core analysis blocks: Structural Analysis, Sampling Design, Surrogate Modeling, and Statistical Analysis. Within each step, specific methodological approaches are applied to serve the corresponding analytical objectives. The sequential steps of the workflow are explained in more detail as follows:

1. *Physical model*: The physical model is used both at the beginning of the workflow and after the uncertainty modeling step, serving different purposes in each case. Initially, a structural analysis is performed to assess the FEM model and conduct a preliminary local sensitivity analysis, which helps to identify critical regions and inform the uncertainty modeling. After defining the uncertainties of the material parameters, the physical model is evaluated again using sampling-based input data. These simulations provide both system responses and updated local sensitivity information, which are essential for building the GEK surrogate model.
2. *Uncertainty modeling*: As with the physical model, the uncertainty modeling step also serves two purposes. Firstly, the generation of a training data set for the surrogate model and secondly, the propagation of uncertainties in the input parameters to the system responses. The initial structural analysis of the physical model and a primary local sensitivity analysis provide information about the number of uncertain parameters and their associated sample space. These specifications serves as the basis for constructing a training data set using LHS. The resulting data set of input values for the material parameters is communicated back to the physical model for evaluation. We use LHS to obtain a space-filling sample of the parameter space on the basis of which the surrogate model is fitted in the complexity reduction step. In the later step of uncertainty quantification, random sampling is used to ensure a thorough investigation of the input uncertainties.
3. *Complexity reduction*: In order to reduce the complexity and the computational load of the physical model, we integrate a surrogate model that emulates the system’s behavior into our analysis workflow. Specifically, a Kriging model is adapted that incorporates the additional information from the local sensitivity analysis, resulting in the GEK model. The data set generated by LHS as part of uncertainty modeling and the corresponding output of the FEM model as part of the physical model are used to fit the GEK model. Furthermore, the partial derivatives of the FEM model outputs with respect to the individual input parameters are given by the sensitivities. These are also computed at the input values of the training data set, resulting in a data set of gradients that is additionally included in the construction of the GEK model.
4. *Uncertainty quantification*: Based on the surrogate model, MC experiments are carried out with randomly selected material parameters according to their uncertainty distribution, which serve to deduce the uncertainty of the quantities of interest.

In the following chapters, the main methodological components of the workflow, namely the FEM Model, Local Sensitivity Analysis, and GEK, are introduced and explained, as they are essential for understanding the proposed workflow and represent its main novel contributions.

### FEM model

#### Physical model

**Fig. 2:**
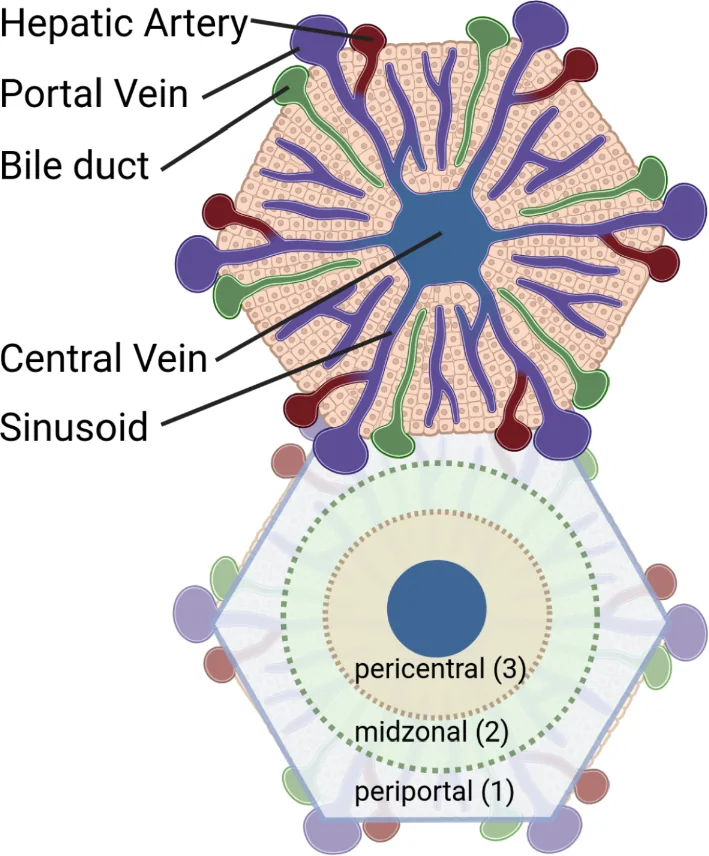
Zonation for liver lobule, adapted from Lambers et al. (2024) and created in BioRender. König (2026) https://BioRender.com/q0bt4ov

Figure 2 illustrates the structure of a liver lobule with the zonation of inflow and outflow areas, which include the periportal and pericentral regions, each with characteristic arrangements of blood vessels and bile ducts.

One critical factor in tumor development is MAFLD, a common condition characterized by excessive fat accumulation in liver cells. This can lead to carcinogenic changes and the subsequent development of HCC. Key external concentrations crucial for fat metabolism, which contribute to MAFLD and tumor development, include free fatty acids, oxygen, and glucose. These substances are transported through the bloodstream by advection, driven by a periportal pressure gradient that decreases from 133 Pa, and are further assisted by diffusion due to concentration gradients. Free fatty acids and glucose, derived from the digestion of dietary fats and carbohydrates respectively, play distinct roles in the body: high levels of free fatty acids can lead to the accumulation of lipid droplets in liver cells, while the consumption of glucose during tumor development highlights its crucial role in cellular metabolism and growth. Conversely, oxygen aids in the oxidation of free fatty acids, thus reducing fat accumulation.

A coupled multiscale and multiphysical approach is employed to simulate the metabolism of the liver lobule considering isotropic permeability with hepatic blood flow. This approach models the complex interactions between the metabolically active cellular level and the vascular systems using the eTPM. The mixture theory (Ateshian and Ricken 2010; Bowen 1980) offers a macroscopic representation of biological tissue by describing it as a system of interacting phases, while allowing the incorporation of microscale particles. The actual structure is homogenized, resulting in a computational model in which different components are averaged, and the physical and chemical properties of the microstructure are represented by these average values. The total description of the mixture body φ is given by summing the individual constituents:

1 $$\begin{aligned} \varphi = \sum _{\alpha =1}^{\kappa } \varphi ^{\alpha } \end{aligned}$$

where $\varphi ^{\alpha }$ represents the individual constituent phases and κ is the number of phases in the mixture. In detail, this approach considers the solid phases: solid liver tissue $\varphi ^{\mathrm S}$, fat tissue $\varphi ^{\mathrm T}$ resulting from fat accumulation and necrotic tissue $\varphi ^{\mathrm C}$ caused by cell injury, and the fluid phase $\varphi ^{\mathrm F}$ representing blood.

Within this framework, the governing equations are summarized in compact form, the full phase-wise formulation is given in Lambers et al. (2024). These equations represent the balance of linear momentum, mass conservation of the individual phases, and transport of chemical species within the mixture.

2 $$\mathbf{0} = {\mathrm{div}\;\mathbf{T}}^{\alpha } ,0 = \left( {\mathrm n^{\alpha } } \right)_{{\mathrm{S}}}^{\prime } + \mathrm n^{\alpha } {\mathrm{tr}}\;{\mathbf{D}}_{{\mathrm{S}}} - \frac{{\hat{\rho }^{\alpha } }}{{\rho ^{{\alpha {\mathrm{R}}}} }},0 = \mathrm n^{\alpha } \left( {\mathrm c^{{\alpha \beta }} } \right)_{\alpha }^{\prime } + \mathrm c^{{\alpha \beta }} \left( {\mathrm n^{\alpha } } \right)_{{\mathrm{S}}}^{\prime } + \mathrm n^{\alpha } {\mathrm{c}}^{{\alpha \beta }} {\mathbf{D}}_{{\mathrm{S}}} \cdot \mathbf{I} - \frac{{\hat{\rho }^{{\alpha \beta }} }}{{\mathbf{M}_{{{\mathrm{mol}}}}^{\beta } }}$$

Here, $\alpha \in \{\mathrm S,\mathrm T,\mathrm C,\mathrm F\}$ denotes the different phases, and β denotes the associated constituents (e.g. nutrients). The variables $\mathrm n^{\alpha }$ represent the volume fractions, $\bf {\mathrm{T}}^{\alpha }$ the partial Cauchy stress tensors, ${\mathbf{D}}_{{\mathrm{S}}}$ the rate of deformation tensor of the solid phase, $\hat{\rho }^{\alpha }$ and $\hat{\rho }^{\alpha \beta }$ denote mass exchange terms, and ${\mathbf{M}}_{{{\mathrm{mol}}}}^{\beta }$ the molar mass of constituent β. Furthermore, $\mathrm c^{\alpha \beta }$ represents transport-related quantities associated with the constituents. The resulting coupled system describes the interaction between mechanical deformation, fluid transport, and metabolic processes within the liver tissue. The primary variables of the formulation are given by

$$\left[ {{\mathbf{u}}^{{\mathrm{S}}} ,\; \mathrm p^{{{\mathrm{FR}}}} ,\;\mathrm n^{{\mathrm{S}}} ,\;\mathrm n^{{\mathrm{T}}} ,\;\mathrm n^{{\mathrm{C}}} ,\;\mu ^{{\alpha \beta }} } \right],$$

where $\mathbf u^{{\mathrm{S}}}$ denotes the displacement of the solid phase, $\mathrm p^{{{\mathrm{FR}}}}$ the fluid pressure, and $\mu ^{\mathrm {\alpha \beta }}$ the chemical potentials associated with the interacting constituents. To close the system of equations, the constitutive behavior of the solid phase is defined via a Helmholtz free energy function assuming a Neo-Hookean material model (cf. 4), from which the stress response is derived. This model is commonly adopted in soft tissue modeling due to its robustness and its ability to capture large deformations (Ricken et al. 2010; Lambers et al. 2024), while its relatively simple structure and limited number of material parameters make it particularly suitable for uncertainty quantification.

#### Boundary value problem

**Fig. 3:**
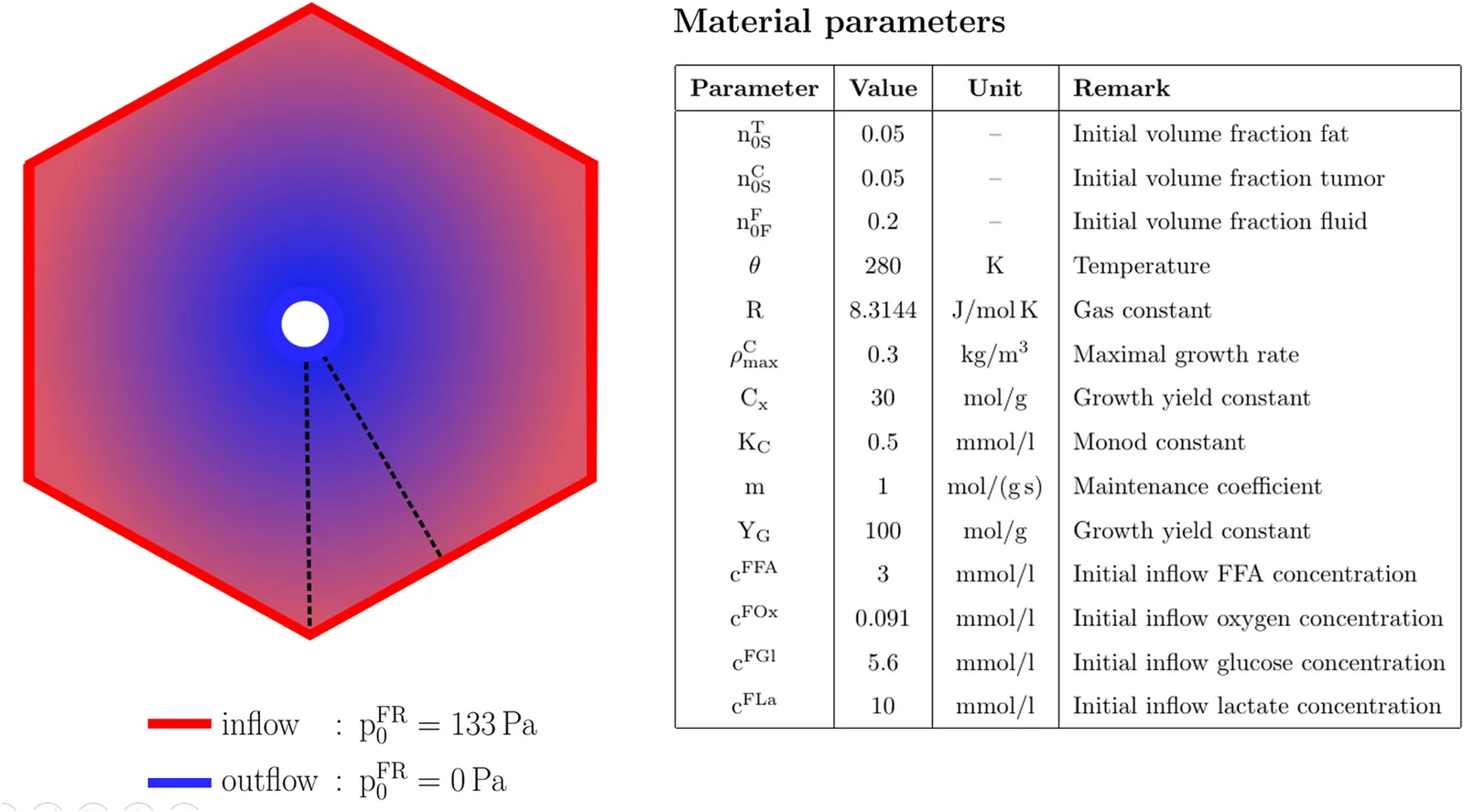
Boundary conditions (left) and material parameters (right) for the numerical study of hepatocellular carcinoma development in liver lobules with pre-existing fat accumulation. The inflow boundary represents the homogenized mixed supply of oxygenated blood from the hepatic artery and nutrient-rich blood from the portal vein, prescribed through effective inflow concentrations (e.g. oxygen, free fatty acids, glucose, and lactate). The outflow boundary corresponds to the central vein

Figure 3 provides a comprehensive overview of the numerical implementation geometry, boundary conditions, and material parameters used to simulate HCC in a liver lobule. The model reduces the geometry of a liver lobule to one-twelfth of its ideal hexagonal shape, employing rotational symmetry to enhance efficiency. Boundary conditions are meticulously established to emulate physiological blood flow and metabolic processes in liver lobules, including realistic inflow at the lobule periphery, and outflow at the central vein. From a numerical perspective, a prescribed pressure is applied at the inflow boundary, while a reference pressure is imposed at the outflow boundary (cf. Figure 3). Symmetry boundary conditions are enforced along the lateral boundaries, and rigid body motion is prevented by appropriate displacement constraints. The simulations are performed using an implicit time integration scheme with a constant time step of Δt=1.0, comprising an initial phase of 400 time steps to establish pre-existing fat accumulation, followed by 75 time steps to simulate tumor progression. The resulting non-linear system is solved using a Newton–Raphson scheme. These conditions are critical to accurately simulate metabolic activities and ensure effective nutrient delivery through a directed blood flow with a specified pressure gradient. The numerical simulation starts with pre-existing fat accumulation and incorporates a critical threshold for fat volume fraction; once this threshold is exceeded, tumor development is initiated and modeled using extended Monod kinetics. Furthermore, the fat metabolism as described in Ricken et al. (2018) is activated so that a constant supply of external concentrations at the inflow lead to metabolism processes which result in further fat accumulation.

#### Uncertain parameters

The development of the mathematical eTPM model, including the derivation of kinematics, balance equations, and the thermodynamically consistent concept, is detailed in the publication of Lambers (2023) and Lambers et al. (2024). In this paper, we highlight the impact of the main uncertain input parameters, resulting from tissue-specific conditions, pathological changes, and experimental limitations. This paper focuses on the following uncertain material parameters:

1. Young’s modulus liver tissue $\mathrm E^{{\mathrm{S}}}$
2. Young’s modulus fat tissue $\mathrm E^{\mathrm T}$
3. Young’s modulus necrotic tissue $\mathrm E^{\mathrm C}$
4. Poisson’s ratio ν
5. Darcy permeability $$\mathrm k_{{\mathrm{D}}}$$
6. Initial solid volume fraction liver tissue $\mathrm n_{{\mathrm{0}}}^{{\mathrm{S}}}$.

The uncertain Young’s modulus $\mathrm E^{\alpha }$ and Poisson’s ratio are fundamental parameters for describing the elastic properties of the material. They are connected to the Lamé constants $\lambda ^{\alpha }$ and $\mu ^{\alpha }$ as follows

3 $$\ \lambda ^{\alpha } = \frac{{\rm E^{\alpha } \nu }}{{(1 + \nu )(1 - 2\nu )}},\;\;\;\;\;\;\;\;\mu ^{\alpha } = \frac{{\rm E^{\alpha } }}{{2(1 + \nu )}}{\mkern 1mu} ,\quad \alpha \in \{ {\rm S},{\rm T},{\rm C}\} .$$

In the eTPM model, these parameters are used to define the Helmholtz free energy for the solid phases $\psi ^{\alpha }$. The Helmholtz free energy function is given by

4 $${\text{ }} \psi^{\alpha } = \frac{1}{{\rho_{0{\mathrm{S}}}^{\alpha } }}\left[ {\lambda ^{\alpha } \frac{1}{2}(\ln {\mathrm{J}}_{\mathrm{S}} )^{2} - \mu ^{\alpha } \ln {\rm J}_{\mathrm{S}} + \frac{1}{2}\mu ^{\alpha } ({\mathrm{tr}}\; \mathbf{C}_{\mathrm{S}} - 3)} \right],\quad \alpha \in \{ {\rm S},{\rm T},{\rm C}\} ,$$

where $\mathrm J_{{\mathrm{S}}}$ is the determinant of the solid deformation gradient and $\mathbf C_{\mathrm{S}}$ is the right Cauchy-Green deformation tensor. The elastic parameters influence the stored energy in the tissue under deformation, as reflected in the logarithmic and tensor-based terms of the Helmholtz free energy for solid phases. Evaluating the uncertainty in stiffness provides insights into how variations in the mechanical properties of liver, fat, and necrotic tissues affect their deformation under applied loads. These uncertainties impact stress and strain distributions in the liver, affecting perfusion, nutrient transport, and fat accumulation, thereby potentially influencing the development of HCC.

Besides mechanical properties, the Darcy permeability is a crucial parameter for describing blood flow characteristics within the liver’s porous structure. The transport of concentrations is governed by fluid flow, which directly affects liver metabolism, fat accumulation, and potentially influences tumor growth. Permeability in biological tissues is subject to significant variability and is difficult to measure reliably in vivo. In contrast to controlled experimental settings, it depends on microstructural features such as porosity, tissue composition, and pathological alterations. Therefore, the Darcy permeability $\mathrm k_{{\mathrm{D}}}$ is treated as an uncertain parameter in this study. The relation between fluid and solid velocity, is determined with the seepage velocity $\mathbf w_{{{\mathrm{FS}}}}$ using the constitutive relationship:

5 $$\mathrm n^{{\mathrm{F}}} {\mathbf{w}}_{{{\mathrm{FS}}}} = {\mathbf{K}}_{{\mathrm{F}}} \left( { - \nabla \lambda } \right),$$

where λ is the pressure and ${\mathbf{K}}_{{\mathrm{F}}}$ is the permeability tensor. This tensor incorporates the Darcy permeability $\mathrm k_{{\mathrm{D}}}$ and the initial solid volume fraction $\mathrm n_{0}^{{\mathrm{S}}}$, both of which are key parameters for modeling fluid transport. The permeability tensor is expressed as:

6 $$\ {\mathbf{K}}_{{\mathrm{F}}} = \mathrm k_{{\mathrm{D}}} \left( {\frac{{\mathrm n^{{\mathrm{F}}} }}{{1 - \mathrm n_{0}^{{\mathrm{S}}} }}} \right)^{{{\mathrm{m}}_{{\mathrm{D}}} }} {\mathbf{M}}^{*} ,$$

therein $\mathrm m_{{\mathrm{D}}}$ characterizes the non-linear dependence of permeability on the fluid fraction, and $\mathbf M^{*}$ captures the anisotropic properties of the permeability tensor. In the present study, an isotropic permeability model is adopted by setting ${\mathbf{M}}^{*} = \mathbf I$.

The uncertain Darcy permeability $\mathrm k_{{\mathrm{D}}}$ directly influences the seepage velocity $\mathbf w_{\mathrm{FS}}$, which determines the perfusion rates within the liver lobule. An increase in $\mathrm {k_D}$ facilitates fluid flow, potentially enhancing nutrient and oxygen transport, while a decrease restricts flow, which may mimic the effects of fibrosis or other structural pathologies. As a result, the accuracy of $\mathrm {k_D}$ is essential for correctly capturing blood perfusion dynamics and their impact on liver metabolism, as well as, fat accumulation and tumor development. Similarly, uncertain initial solid volume fraction $\mathrm n_0^{\mathrm{S}}$ plays a crucial role in defining the initial void space available for fluid transport. It affects the permeability tensor by scaling the influence of the fluid volume fraction $\mathrm n^{\mathrm{F}}$ in the non-linear term.

In the eTPM framework, the initial solid volume fraction $\mathrm n_0^{\mathrm{S}}$ is a fundamental parameter that governs the distribution of phases within the tissue, influencing both mechanical properties and transport phenomena. Uncertainties in $\mathrm n_0^{\mathrm{S}}$ affect pore space, permeability, fluid pathways, pressure gradients, and nutrient exchange at the lobule scale. Its interaction with the diffusive transport coefficient $\mathrm {k_D}$ and the permeability tensor $\textbf{M}^*$ further modulates the directionality of fluid flow. These coupled effects highlight the importance of accounting for structural and flow-related uncertainties to ensure the model’s predictive reliability. Moreover, $\mathrm n_0^{\mathrm{S}}$ plays a key role in determining the total volume V and mass M of the tissue mixture. For example, in its current configuration the following holds:

7 $$\begin{aligned} \mathrm V&= \int _{\mathrm B_{\mathrm s}} \mathrm{dv} = \sum _{\alpha =1}^{\kappa } \mathrm V^{\alpha } = \int _{\mathrm B_{\mathrm s}} \sum _{\alpha =1}^{\kappa } \mathrm{dv}^{\alpha } = \int _{\mathrm B_{\mathrm s}} \sum _{\alpha =1}^{\kappa } \mathrm n^{\alpha } \mathrm{dv} \end{aligned}$$

8 $$\begin{aligned}\mathrm M&= \sum _{\alpha =1}^{\kappa } \mathrm M^{\alpha } = \int _{\mathrm B_{\mathrm s}} \sum _{\alpha =1}^{\kappa } \rho ^{\alpha } \mathrm{dv}\,. \end{aligned}$$

In this context, $\mathrm{dv}^{\alpha }$ represents the differential volume of phase α, while $\mathrm{dv}$ stands for the differential total volume. The term $\mathrm V^{\alpha }$ denotes the volume of phase α, and $\mathrm{n}^{\alpha }$ is the volume fraction of phase α. The body space is represented by $\mathrm {B_s}$. Additionally, $\mathrm M^{\alpha }$ refers to the mass of phase α, and $\rho ^{\alpha }$ signifies the density of phase α.

The eTPM-based FEM model employed in this study is not newly developed here, but builds upon a previously derived and numerically verified multiscale liver framework. In earlier work, selected model components and predicted trends were validated against experimental, histological, and clinical reference data for perfusion, fat zonation, necrosis, and post-resection hemodynamics (Lambers et al. 2024; Lambers 2023). The novelty of this work lies in the integration of sensitivity analysis, surrogate modeling, and uncertainty quantification within this established framework. The present contribution therefore assumes this established model as a baseline and focuses on uncertainty propagation and surrogate-based analysis.

By incorporating these uncertain parameters into the numerical model, their impact can be systematically evaluated. Sensitivity analyses can identify how variations of the material parameters propagate through the model, affecting key outputs such as blood perfusion, nutrient transport, and metabolic activity. Understanding these effects is critical for developing more reliable liver simulations that accurately reflect physiological and pathological conditions.

### Local sensitivity analysis

Local sensitivity analysis provides crucial insights into which parameters are most critical, enabling more efficient and targeted model refinement and uncertainty quantification. In the context of FEM-based simulations of the human liver, this analysis is particularly valuable due to the complex interplay of biological and physical processes. Using the central finite difference method, each parameter is systematically varied within a small range around its nominal value, and the resulting changes in the output are determined as follows

9 $$f^{\prime}(x) \approx \frac{{f(x + \delta ) - f(x - \delta )}}{{2\delta }}{\mkern 1mu} ,$$

where *f*() refers to the FEM model, *x* are the material parameters and δ is a small perturbation.

### Gradient-Enhanced Kriging

GEK is a technique that improves the accuracy of Kriging models by incorporating gradient information along with the function values. This method was designed to enable fast predictions of complex and computationally intensive models (Morris et al. 1993). The objective of this application is to predict the blood flow velocity and the tumor volume fraction in the liver considering uncertain input parameters. The GEK method is employed to approximate the time-consuming and complex numerical eTPM model, yet allow an accurate prediction of the solutions of interest. Within this contribution, the ordinary GEK model is adapted, which is briefly introduced below. For further descriptions of the GEK model, please also refer to Zimmermann (2013) and van Meegen and Ickstadt (2026).

In the following, consider the output of the computer model *f*(*x*) with the vector of inputs $x = (x_1, \dots , x_d)^\top \in \mathbb {R}^d$, e.g. the material parameters. With the ordinary GEK approach, it is assumed that the output of the computer model is modeled as

10 $$\begin{aligned} f(x) = \beta + z(x), \end{aligned}$$

where β is an unknown constant mean and *z*(*x*) is a Gaussian process with $\mathrm{E}(z(x)) = 0$ and $\mathrm{Cov}(z(x), z(x')) = \sigma ^2 \phi (x,x')$. For the correlation function, the Matérn $\frac{3}{2}$ kernel is chosen, i.e.

11 $$\begin{aligned} \phi (x,x') = \prod _{k = 1}^d \left( 1 + \frac{\sqrt{3}|x_{k} - x_{k}'|}{\theta _k}\right) \exp \left( -\frac{\sqrt{3}|x_{k} - x_{k}'|}{\theta _k}\right) \end{aligned}$$

with unknown hyperparameters $\theta _k>0$ for all k=1,⋯,d.

Consider a design of inputs $x_1, \dots , x_n$, where $x_i = (x_{i1}, \dots , x_{id})^\top \in \mathbb {R}^d$, denotes the *i*-th input vector, i=1,⋯,n. For this design, the computer model is executed and the relevant outputs $y_i = f(x_i) \in \mathbb {R}$ and the corresponding gradients $\nabla y_i = \nabla f(x_i) \in \mathbb {R}^d$ are obtained. The collection of output and gradients are arranged as $\mathbf y = (y_1,\dots , y_n, \nabla y_1^\top ,\dots , \nabla y_n^\top )^\top \in \mathbb {R}^{n +nd}$. Under the model in (10), $\textbf{y}$ is multivariate normally distributed with mean $\mathbf F\beta$. Here, $\mathbf F = (\mathbf 1_{{n}}^{{ \top }} ,\mathbf 0_{{{nd}}}^{{ \top }} )^{{ \top }} \in \mathbb{R}^{{{n + nd}}}$ is the model matrix with derivatives, where $\mathbf{1}_n$ is an n×1 vector of ones and $\mathbf{0}_{nd}$ is the zero vector with *nd* entries. The covariance matrix of $\textbf{y}$ is given by $\sigma ^2 \textbf{R}$ with

$$\begin{aligned} \mathbf R = \begin{pmatrix} r_{x_1, x_1} & \cdots & r_{x_1, x_n} & \frac{\partial r_{x_1,x_1}}{\partial x_1}^\top & \cdots & \frac{\partial r_{x_1,x_n}}{\partial x_n}^\top \\ \vdots & \ddots & \vdots & \vdots & \ddots & \vdots \\ r_{x_n, x_1} & \cdots & r_{x_n, x_n} & \frac{\partial r_{x_n, x_1}}{\partial x_1}^\top & \cdots & \frac{\partial r_{x_n, x_n}}{\partial x_n}^\top \\ \frac{\partial r_{x_1,x_1}}{\partial x_1} & \cdots & \frac{\partial r_{x_n, x_1}}{\partial x_1} & \frac{\partial ^2 r_{x_1, x_1}}{\partial ^2 x_1} & \cdots & \frac{\partial ^2 r_{x_1, x_n}}{\partial x_1 \partial x_n} \\ \vdots & \ddots & \vdots & \vdots & \ddots & \vdots \\ \frac{\partial r_{x_1, x_n}}{\partial x_n} & \cdots & \frac{\partial r_{x_n, x_n}}{\partial x_n} & \frac{\partial ^2 r_{x_n, x_1}}{\partial x_n \partial x_1} & \cdots & \frac{\partial ^2 r_{x_n, x_n}}{\partial ^2 x_n} \end{pmatrix} \in \mathbb {R}^{(n + nd) \times (n + nd)}, \end{aligned}$$

thereby is $\frac{\partial r_{x_i,x_j}}{\partial x_i} = \left( \frac{\partial \phi (x_i, x_j)}{\partial x_{ik}} \right) _{1 \le k \le d} \in \mathbb {R}^d$ and $\frac{\partial ^2 r_{x_i, x_j}}{\partial x_i \partial x_j} = \left( \frac{\partial \phi (x_i, x_j)}{\partial x_{ik} \partial x_{jl}}\right) _{1 \le k,l \le d} \in \mathbb {R}^{d \times d}$ as well as $r_{x_i,x_j} = \phi (x_i, x_j)$ for i,j=1,⋯,n.

For an unevaluated vector of inputs *x*, the objective is to find a prediction $\widehat{f}(x)$ of the computer output *f*(*x*). For this purpose, an arbitrary linear predictor of the form $\mathbf w^\top \textbf{y}$ is considered. The weights $\mathbf w \in \mathbb {R}^{n + nd}$ are obtained by minimizing the mean squared error of the linear predictor subjected to the unbiasedness constraint $\mathbf F^\top \textbf{w} = 1$. This results in the best linear unbiased predictor

12 $$\widehat{f}(x) = \widehat{\beta } + \mathbf r(x)^{{ \top }} \mathbf R^{{ - 1}} (\mathbf y - \mathbf F\widehat{\beta }),$$

where $\mathbf r(x) = (r_{x,x_1}, \dots , r_{x,x_n}, \frac{\partial r_{x,x_1}}{\partial x_1}^\top , \dots , \frac{\partial r_{x,x_n}}{\partial x_n}^\top )^\top \in \mathbb {R}^{n + nd}$ is the auto- and cross-correlation vector and $\widehat{\beta } = (\mathbf F^\top \mathbf R^{-1} \mathbf F)^{-1} \mathbf F^\top \mathbf R^{-1} \mathbf y$ is the generalized least square estimator of β. The estimators of the parameters $\sigma ^2$ and $\theta _1,\dots , \theta _d$ are determined using the maximum likelihood method.

## Application workflow and results

The workflow illustrated in Fig. 1 outlines the integration of the FEM-based liver simulation developed in Ricken et al. (2010, 2018), Lambers (2023), and Lambers et al. (2024) within the framework of the eTPM. It incorporates uncertain material parameters and guides the reader through the main methodological components of the analysis. This chapter introduces a specific boundary value problem as an application example and systematically walks through each step of the workflow.

The application focuses on simulating the progression of HCC, considering pre-existing fat accumulation as a key risk factor for tumor development. This study investigates the impact of HCC on blood perfusion within a liver lobule, with particular emphasis on two critical solution variables: tumor volume fraction $\mathrm n^{\mathrm{C}}$ and blood velocity $\mathbf w_{\mathrm{FS}}$. Given the inherent uncertainties in the model and its underlying assumptions, an uncertainty analysis is essential to account for variability in the material parameters and to ensure the reliability of the simulation results.

### Local sensitivity analysis

The local sensitivity analysis is used to identify which area of the liver lobule should be examined further within the framework of uncertainty quantification. Therefore, we analyze the impact of all uncertain material parameters on two key solution variables: tumor volume fraction $\mathrm n^{\mathrm{C}}$ and blood velocity $\mathbf w_{\mathrm{FS}}$ throughout the entire liver lobule. We conduct a total of 50 simulations using a uniform sampling function with predefined minimum and maximum values, see Table 1, enabling the evaluation of parameter sensitivities.

**Fig. 4:**
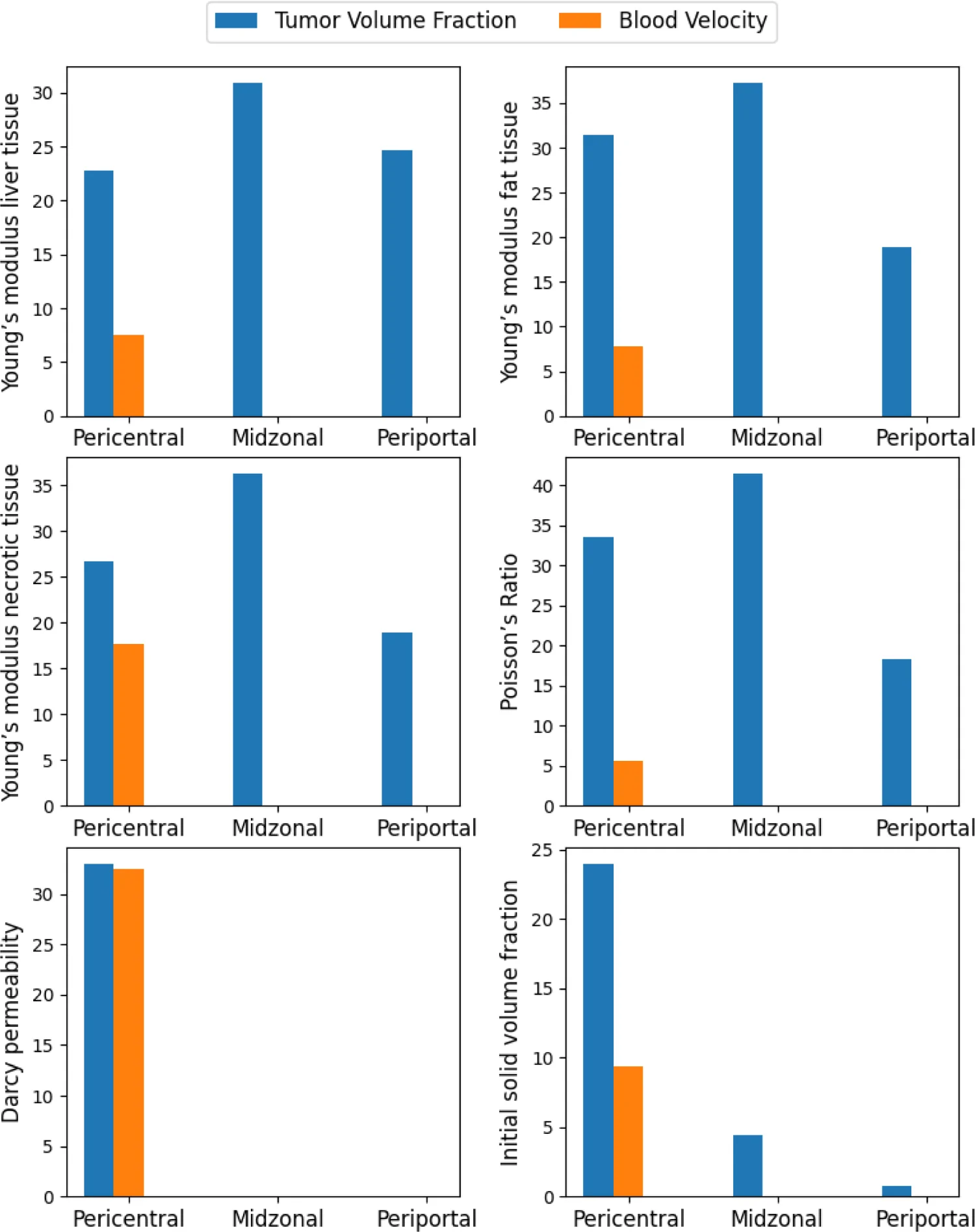
Percentage of maximum sensitivity of the solution of interest: tumor volume fraction (blue) and blood velocity (orange), across the pericentral, midzonal, and periportal regions of the liver lobule

Figure 4 illustrates the percentage of maximum sensitivity for two key output parameters, tumor volume fraction (blue) and blood velocity (orange), across the periportal, midzonal, and pericentral regions of the liver lobule. Blood velocity shows a zonal pattern, with maximum sensitivities occurring most frequently in the pericentral region. In contrast, no consistent zonal trend is observed for the tumor volume fraction with respect to the uncertain stiffness parameters. For variations in Darcy permeability and the initial solid volume fraction, the locations of maximum sensitivity are also predominantly found in the pericentral region.

**Fig. 5:**
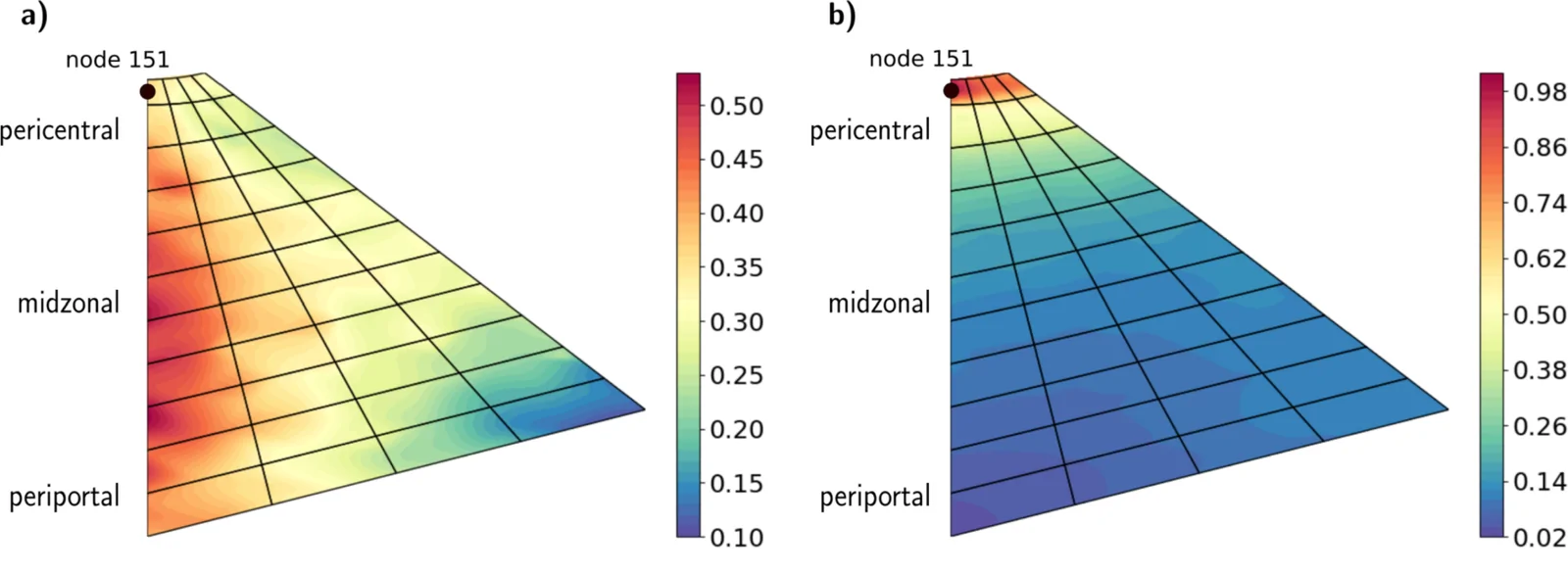
Spatial distribution of the normalized and averaged sensitivities for the solutions of interest within one-twelfth of the idealized hexagonal liver lobule, based on 50 simulations covering the minimum and maximum bounds of all uncertain parameters. a) Tumor volume fraction and b) blood velocity. The periportal, midzonal, and pericentral regions are indicated along the sinusoidal direction

In Fig. 5, the normalized and averaged sensitivities are spatially resolved within the liver lobule, illustrating sensitivities for the solution variables. Sensitivities for blood velocity are predominantly observed in the pericentral area. Overall, the results indicate that the pericentral region of the liver is most frequently associated with the locations of maximum local sensitivity. The robustness of the identified sensitivity patterns with respect to the number of samples is further investigated in Appendix A.1. Node 151 is located in the pericentral region and is used as a representative point for the subsequent analysis. Additional nodes within the pericentral region were evaluated, confirming qualitatively similar sensitivity patterns.

### Uncertainty modeling

The uncertain input parameters of Sect. 2.2 are treated consistently for both surrogate modeling and uncertainty analysis, utilizing the same statistical distributions for each input parameter. Each parameter has its distribution, mean, and standard deviation, with lognormal distributions for the Young’s modulus parameters and Darcy permeability, and uniform distributions for Poisson’s ratio and solid volume fraction. The uncertain input parameters and their probability distributions are summarized in Table 1. The mean values correspond to the baseline deterministic parameter set of the FEM model. Lognormal distributions are assigned to the material stiffness and permeability parameters. Uniform distributions are used for volume fractions and geometric parameters. The parameter ranges are chosen based on physiologically plausible values reported in the literature and previous modeling studies (Lambers et al. 2024; Lambers 2023).

**Table 1:** Overview of the uncertain input parameters in the FEM model with corresponding uncertainty distribution, as well as their expected value, standard deviation (Std.), coefficients of variation (CoV), and maximum and minimum values

Parameter [Unit]	Distribution	Mean	Std	CoV	Min	Max
${\mathrm{E}}^{{\mathrm{S}}}$ [Pa]	Lognormal	9550	2800	0.293	3000	28000
${\mathrm{E}}^{{\mathrm{T}}}$ [Pa]	Lognormal	9550	2800	0.293	3000	28000
${\mathrm{E}}^{{\mathrm{C}}}$ [Pa]	Lognormal	9550	2800	0.293	3000	28000
ν [–]	Uniform	0.190	0.052	0.273	0.100	0.280
${\mathrm{k}}_{{\mathrm{D}}}$ [m/s]	Lognormal	$5.66 \times 10^{-9}$	$1.49 \times 10^{-9}$	0.263	$2.00 \times 10^{-9}$	$1.50 \times 10^{-8}$
${\mathrm{n}}_0^{{\mathrm{S}}}$ [–]	Uniform	0.450	0.202	0.449	0.100	0.800

### Complexity reduction via surrogate modeling

Based on the findings of the local sensitivity analysis in Sect. 3.1, we construct separate surrogate models for the blood velocity and the tumor volume fraction in node 151, as this node was identified in the pericentral region. The surrogate model takes into account the uncertain inputs parameters described in Sect. 3.2, while the remaining material parameters are fixed at their baseline values for the following analyses, see Fig. 3.

The procedure to construct a surrogate model for the blood velocity or the tumor volume fraction in node 151, i.e. a GEK model, includes several key steps. First, a maximin LHS on $[0, 1]^d$ with n=15d observations is generated, where d=6 is the number of input parameters (McKay et al. 1979; Stein 1987). This is done with the R (R Core Team 2025) package lhs (Carnell 2024). The inputs from the LHS are then mapped to the corresponding physical range between the minimum and maximum values of the parameters, see Table 1, using a linear transformation. This results in a set of design points $\{x_i = (\mathrm E_i^{\mathrm{S}}, \mathrm E_i^{\mathrm{T}},\mathrm E_i^{\mathrm{C}}, \nu _i,\mathrm k_{\mathrm{D}i},\mathrm n_{0i}^{\mathrm{S}})^\top \}_{i = 1,\dots , 90}$. These transformed inputs are used to evaluated the FEM model introduced in Sect. 2.2 to obtain a set of outputs. Next, a GEK model is created based on the outputs and the results of the local sensitivity analysis, cf. Sections 2.3 and 2.4. For numerical stability, the GEK models are trained on the normalized inputs generated by LHS, instead of the transformed samples. For this purpose, the derivatives are transformed accordingly so that they are defined with respect to the uniform input space. Thus, the GEK models predict blood flow velocity and tumor volume fraction based on the normalized inputs, respectively. A comparison with GEK models based on the transformed inputs shows similar predictions. However, the resulting correlation matrix $\textbf{R}$ is ill-conditioned compared to that from the model based on the normalized inputs, making later ones numerically more stable. Furthermore, we compared GEK models with Gaussian, Matérn $\frac{5}{2}$, and Matérn $\frac{3}{2}$ correlation function. Since all models yield similar results, the presentation of the results is limited to the GEK models with the Matérn $\frac{3}{2}$ correlation function. The GEK models were constructed using the R package gek (van Meegen 2026).

**Fig. 6:**
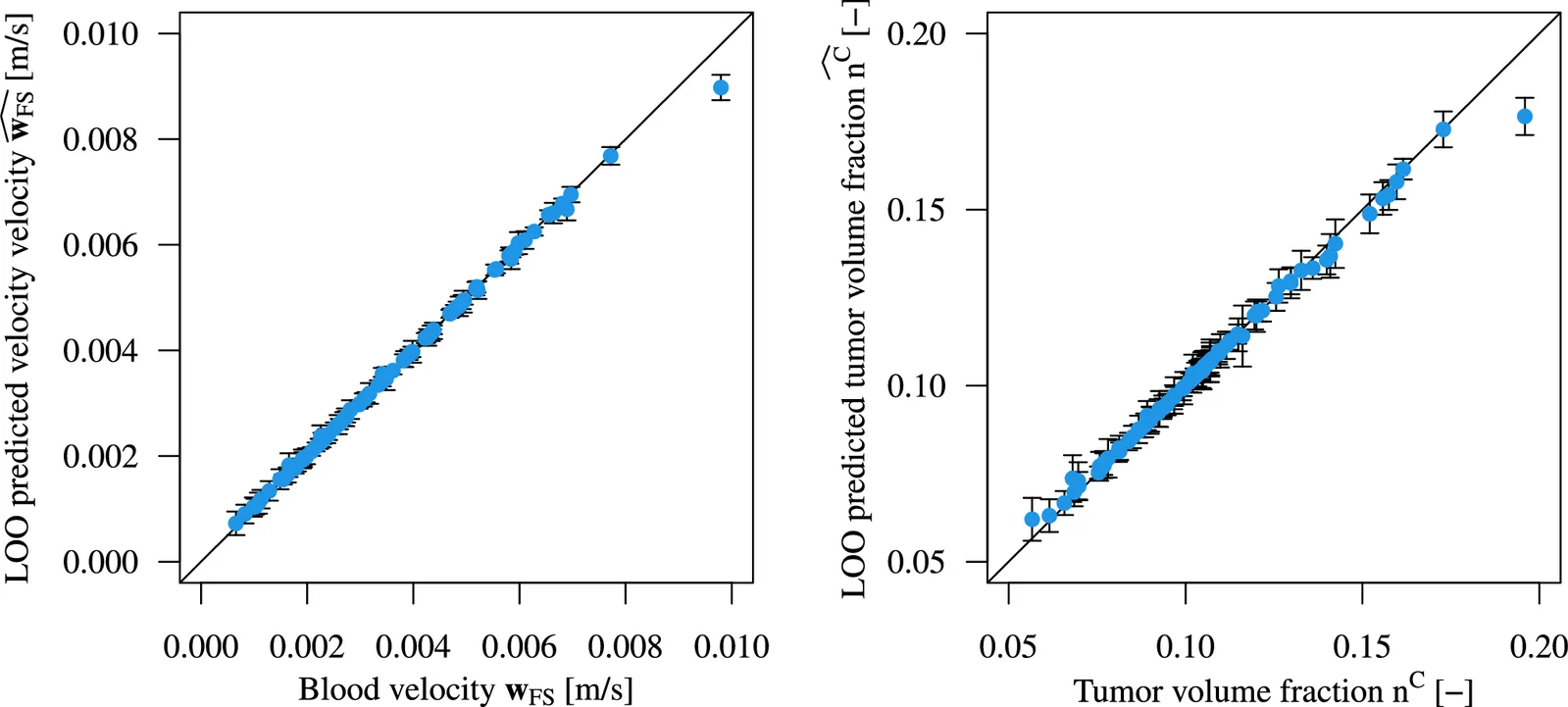
Leave-One-Out (LOO) cross-validated emulator predictions of the blood velocity (left) and tumor volume fraction (right) in node 151 with corresponding 95% confidence intervals

In order to validate the prediction accuracy of the resulting GEK models and to minimize the computational effort, especially for the FEM model, we perform a Leave-One-Out (LOO) cross-validation (Dubrule 1983; Loeppky et al. 2009). LOO cross-validation is a suitable validation technique and standard method, especially when only a few observations are available and generating new observations is computationally expensive. In this procedure, each observation is removed once from the data set and the GEK model is estimated on the remaining n-1 observations. For the observation that was not included in the training data set, a prediction is determined using the emulator. In Fig. 6, the LOO predictions of the emulator together with the 95% confidence intervals are plotted against the solutions of interest calculated by the eTPM model. The predictions are close to the reference solution, with narrow confidence intervals.

In addition, we consider the proportion of outcomes that lie within the 95% confidence intervals of the GEK model, i.e.

13 $$\mathrm P{_{\mathrm{CI}}(95\%)} = \frac{1}{n}\sum_{i = 1}^n \textbf{1}\left\{ f(x^*_i) \in \left[ \widehat{f}_{\mathrm{0.025}}(x^*_i), \widehat{f}_{\mathrm{0.975}}(x^*_i)\right] \right\} ,$$

the mean length of the 95% confidence intervals

14 $$\mathrm L_{{{\mathrm{CI}}}} (95\% ) = \frac{1}{n}\sum\limits_{{i = 1}}^{n} {\left( {\widehat{f}_{{{\mathrm{0}}.{\mathrm{975}}}} (x_{i}^{*} ) - \widehat{f}_{{{\mathrm{0}}.{\mathrm{025}}}} (x_{i}^{*} )} \right)} ,$$

and the root mean squared error

15 $${\mathrm{RMSE}} = \sqrt {\frac{1}{n}\sum\limits_{{i = 1}}^{n} {\left( {f(x_{i}^{*} ) - \widehat{f}(x_{i}^{*} )} \right)^{2} } }$$

as diagnostics (Bastos and O’Hagan 2009; Gu and Berger 2016). Here, $$\widehat{f}(x^*_i)$$ is the LOO prediction at the i-th held-out input $$x^*_i$$ for the corresponding simulator output $$f(x^*_i)$$. Furthermore, $$\textbf{1}\{\cdot \}$$ is an indicator function that is 1 if $$f(x^*_i) \in \left[ \widehat{f}_{\mathrm{0.025}}(x^*_i), \widehat{f}_{\mathrm{0.975}}(x^*_i)\right]$$ and 0 otherwise, where $$\widehat{f}_{0.025}(x^*_i)$$ and $$\widehat{f}_{0.975}(x^*_i)$$ denote the lower and upper limits of the 95% confidence interval for the LOO prediction $$\widehat{f}(x^*_i)$$, respectively. For the blood velocity, we obtain $$\mathrm P_{\mathrm{CI}}(95\%) = 0.967$$, which is slightly above the nominal level of 95%. However, the mean length of the confidence intervals is relatively small with $$\mathrm L_{\mathrm{CI}}(95\%) = 2.564 \times 10^{-4}$$ and the resulting RMSE is $$9.668 \times 10^{-5}$$. In the case of the tumor volume fraction, $$\mathrm P_{\mathrm{CI}}(95\%) = 
0.978$$ and $$\mathrm L_{\mathrm{CI}}(95\%) = 7.620 \times 10^{-3}$$. The RMSE is $$2.488 \times 10^{-3}$$.

**Fig. 7:**
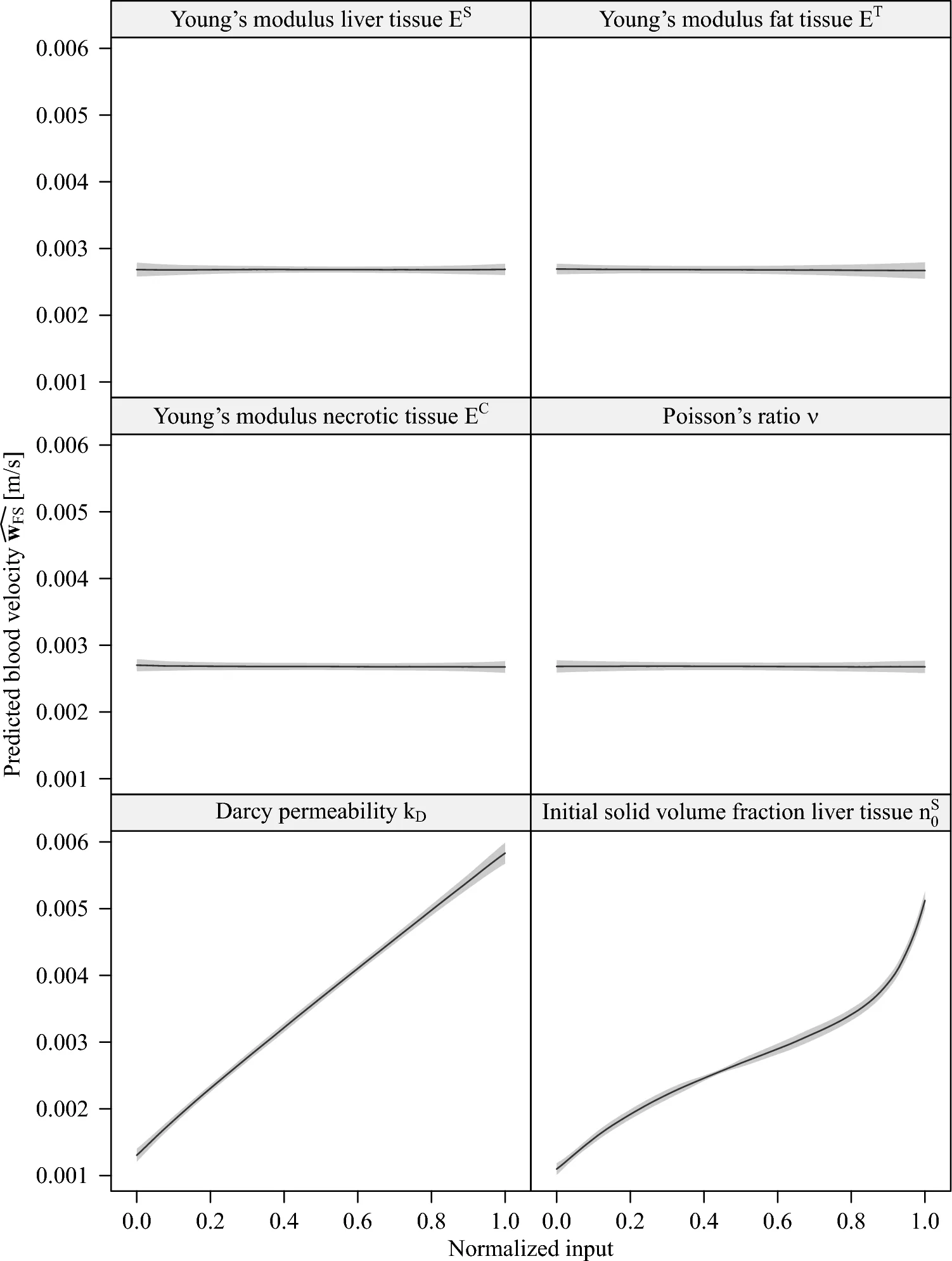
Predicted blood flow velocity in node 151 of the Gradient-Enhanced Kriging model as a function of one normalized input parameter (solid line) with associated 95% uncertainty intervals (shaded area) in each case. Only one input changes at a time, while all other inputs are fixed at their mean value

**Fig. 8:**
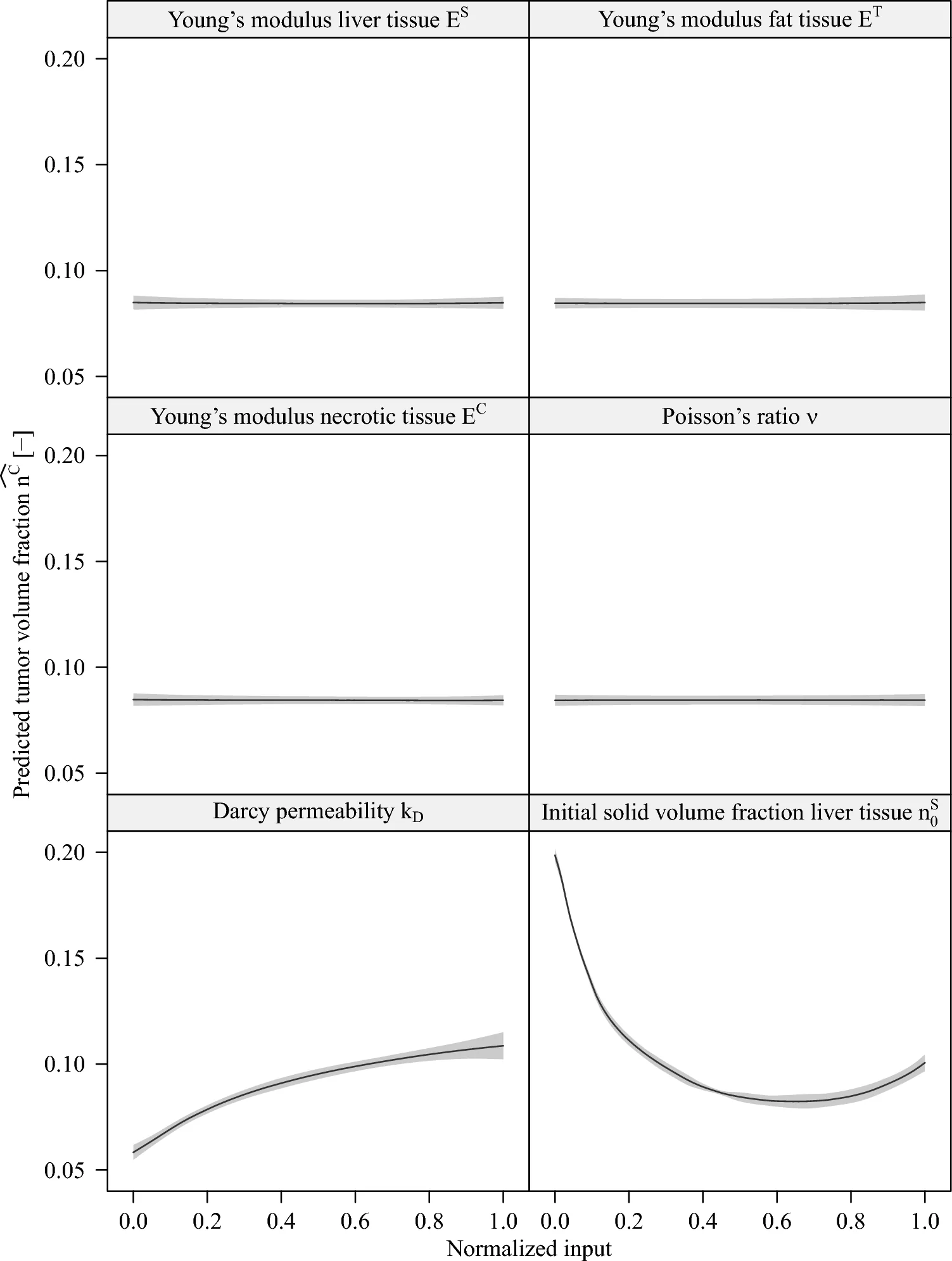
Predicted tumor volume fraction in node 151 of the Gradient-Enhanced Kriging model as a function of one normalized input parameter (solid line) with associated 95% uncertainty intervals (shaded area) in each case. Only one input changes at a time, while all other inputs are fixed at their mean value

Figures 7 and 8 illustrate the predicted blood flow velocity and tumor volume fraction of the GEK model as a function of one normalized input parameter, along with the associated 95% uncertainty intervals. This gives an initial insight into how sensitive the solution variables are to changes in the input parameters. The results show that the elasticity parameters have no observable influence on the tumor volume fraction $\mathrm n^{\mathrm{C}}$ and the blood velocity $\mathbf w_{\mathrm{FS}}$ in the pericentral region. This observation is also obtained in additional parametric studies using the underlying eTPM-based model. In contrast, the parameters with the strongest influence are the Darcy permeability $\mathrm k_{\mathrm{D}}$ and the initial solid volume fraction $\mathrm n_0^{\mathrm{S}}$. Increasing Darcy permeability $\mathrm k_{\mathrm{D}}$ is associated with higher values of $\mathrm n^{\mathrm{C}}$ and increased blood velocity. The relationship between the solution variables and the initial solid volume fraction $\mathrm n_0^{\mathrm{S}}$ exhibits a non-linear dependence, particularly for $\mathrm n^{\mathrm{C}}$. At lower values of $\mathrm n_0^{\mathrm S}$, increasing $\mathrm n_0^{\mathrm{S}}$ is associated with reduced tumor growth, while at higher values, an increase in $\mathrm n^{\mathrm{C}}$ is observed.

### Uncertainty quantification

Following the construction of the GEK model, MC methods are conducted to obtain the uncertainty distribution of the blood flow velocity and tumor volume fraction. The MC approach takes into account not only the uncertainty of the input parameters, but also the uncertainty of the surrogate model about the computer code, i.e. code uncertainty. In this analysis, a total of $n_{\mathrm{MC}} = 5 \times 10^5$ random inputs are generated according to the parameter distributions, see Table 1, under the assumption of pairwise independence. These random inputs are, in turn, normalized and used as inputs. To capture the code uncertainty within this simulation, $n_{{{\mathrm{sim}}}} = 1000$ conditional process paths are drawn on the basis of the GEK models and given the generated MC samples. The convergence of the MC simulations is evaluated separately to ensure that a total number of $n_{\mathrm{MC}} = 5 \times 10^5$ MC samples and $n_{\mathrm{sim}} = 1000$ generated process paths are sufficient to obtain stable estimates within narrow confidence bounds, see Appendix A.2.

**Fig. 9:**
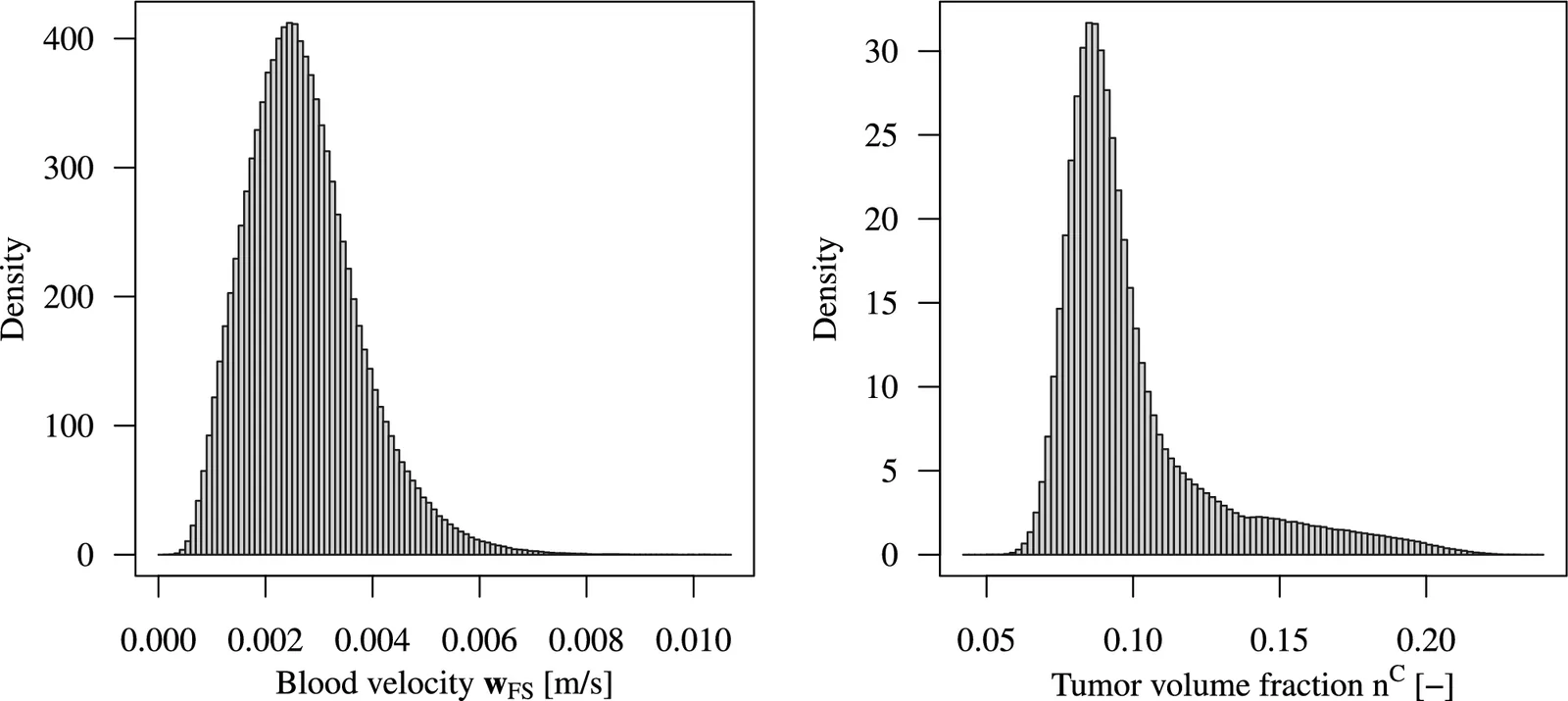
Histograms of the uncertainty distributions for blood flow velocity (left) and tumor volume fraction (right) in node 151, obtained via Monte Carlo based on the Gradient-Enhanced Kriging model

Figure 9 presents the resulting uncertainty distribution of the blood flow velocity and the tumor volume fraction in node 151 obtained through the MC approach as histograms. Here, both the uncertainty distribution of the blood velocity and the tumor volume fraction are rather right-skewed. However, the uncertainty distribution of the tumor volume fraction exhibits a right co-located heavy tail, with higher values observed within the sampled realizations. The observed variability is associated with the non-linear dependence on the initial solid volume fraction $\mathrm n_0^{\mathrm{S}}$.

Additional estimated descriptive statistics of the uncertainty distribution of the blood velocity $\mathbf w_{\mathrm{FS}}$ and the tumor volume fraction $\mathrm n^{\mathrm{C}}$ are summarized in Table 2. For almost all descriptive statistics, we obtain relatively small standard deviations and narrow credibility intervals. This suggests that the code uncertainty is low and that the uncertainty in the outcomes is mainly due to the uncertainty in the inputs.

**Table 2:** Estimated statistics of the uncertainty distribution of the blood velocity $\mathbf w_{\mathrm{FS}}$ [m/s] and the tumor volume fraction $\mathrm n^{\mathrm{C}}$ [−] in node 151 and corresponding standard deviations (Std.) and 95% credibility intervals (CI)

	blood velocity $\mathbf w_{\mathrm{FS}}$				tumor volume fraction $\mathrm n^{\mathrm{C}}$			
	Estimate	Std	95% CI		Estimate	Std	95% CI	
Mean	$2.710 \times 10^{-3}$	$1.136 \times 10^{-6}$	$2.707 \times 10^{-3}$	$2.712 \times 10^{-3}$	$9.968 \times 10^{-2}$	$3.261 \times 10^{-5}$	$9.961 \times 10^{-2}$	$9.974 \times 10^{-2}$
Median	$2.593 \times 10^{-3}$	$1.510 \times 10^{-6}$	$2.589 \times 10^{-3}$	$2.595 \times 10^{-3}$	$9.106 \times 10^{-2}$	$4.448 \times 10^{-5}$	$9.098 \times 10^{-2}$	$9.115 \times 10^{-2}$
Std	$1.059 \times 10^{-3}$	$8.766 \times 10^{-7}$	$1.058 \times 10^{-3}$	$1.061 \times 10^{-3}$	$2.705 \times 10^{-2}$	$2.455 \times 10^{-5}$	$2.701 \times 10^{-2}$	$2.710 \times 10^{-2}$
Min	$1.807 \times 10^{-4}$	$4.180 \times 10^{-5}$	$8.474 \times 10^{-5}$	$2.513 \times 10^{-4}$	$5.089 \times 10^{-2}$	$1.793 \times 10^{-3}$	$4.680 \times 10^{-2}$	$5.366 \times 10^{-2}$
Max	$1.031 \times 10^{-2}$	$1.085 \times 10^{-4}$	$1.012 \times 10^{-2}$	$1.054 \times 10^{-2}$	$2.324 \times 10^{-1}$	$1.702 \times 10^{-3}$	$2.292 \times 10^{-1}$	$2.358 \times 10^{-1}$
Skewness	0.793	$2.835 \times 10^{-3}$	0.788	0.799	1.834	$3.283 \times 10^{-3}$	1.827	1.840
Kurtosis	4.130	$8.075 \times 10^{-3}$	4.114	4.146	6.254	$1.355 \times 10^{-2}$	6.229	6.280

## Discussion and summary

By integrating rigorous uncertainty quantification into the previously deterministic Finite Element Method (FEM)-based extended Theory of Porous Media (eTPM) liver model (Ricken et al. 2010, 2018; Lambers et al. 2024). This paper presents a comprehensive framework for propagating uncertain material parameters and evaluating their effects on the numerical solution, cf. Fig. 1. The high complexity of the numerical model necessitated a preliminary local sensitivity analysis and the development of a surrogate model to enable computationally efficient uncertainty quantification. The uncertainty analysis is based on a generalized workflow that can be readily transferred to other applications involving FEM-based models.

The workflow is applied to simulate the progression of hepatocellular carcinoma (HCC), incorporating fat accumulation as a key risk factor. The first objective of the study is to identify critical parameters through a local sensitivity analysis. The simulation provides time- and space-resolved numerical solutions within a liver lobule, modeled as one-twelfth of an idealized hexagonal structure. Physiologically realistic boundary conditions are defined to represent blood flow and metabolism, beginning with pre-existing fat accumulation. Once a critical fat threshold is exceeded, tumor growth is initiated and modeled using extended Monod kinetics. A local sensitivity analysis is conducted to determine which regions of the lobule warrant further investigation. Based on 50 simulations using uniformly sampled uncertain material parameters, the influence on two key solution variables, blood velocity $\mathbf w_{\mathrm{FS}}$ and tumor volume fraction $\mathrm n^{\mathrm{C}}$, is evaluated. The analysis reveals that the pericentral zone is the most sensitive region and is therefore selected as the spatial region of interest for subsequent investigation. The increased sensitivity observed in the pericentral region may be attributed to significant changes in liver conditions due to tumor influence and increased fat accumulation, as well as alterations in microperfusion (Ricken et al. 2018; Lambers 2023). Furthermore, this region exhibits complex non-linear behavior driven by the strong coupling of advection, diffusion, and metabolic processes, which may amplify sensitivities. These findings are consistent with previous studies, highlighting the critical interplay of physiological and pathological factors influencing liver function.

Guided by these findings, the second objective is to employ a Gradient-Enhanced Kriging (GEK) surrogate model. Based on the local sensitivity results, the GEK model is constructed for blood velocity and tumor volume fraction at a representative pericentral node. Using Latin Hypercube Sampling with six uncertain input parameters, the FEM model is evaluated and the GEK is trained. Model accuracy is validated through Leave-One-Out cross-validation, showing good agreement with the FEM results and narrow confidence intervals.

As the third objective, the uncertainty in material parameters is systematically evaluated. The results show that the elasticity parameters have little influence on blood velocity and tumor volume fraction in the pericentral region. This contrasts with physiological expectations and suggests that the model may underrepresent mechano-biological coupling under the current assumptions. These findings motivate further investigation and potential refinement of the eTPM formulation to verify and better capture such coupling mechanisms. In contrast, $\mathbf w_{\mathrm{FS}}$ and $\mathrm n^{\mathrm{C}}$ respond strongly to variations in Darcy permeability $\mathrm k_{{\mathrm{D}}}$ and initial solid volume fraction $\mathrm n_{{\mathrm{0}}}^{{\mathrm{S}}}$, exhibiting non-linear effects related to tissue porosity, blood flow, and metabolite transport.

Finally, the last objective addresses the uncertainty distribution in the numerical simulation results assessing the impact of HCC in a liver lobule. The eTPM model outputs, including blood flow velocity $\mathbf w_{\mathrm{FS}}$ and tumor volume fraction $\mathrm n^{\mathrm{C}}$, exhibit right-skewed distributions under variation of the input parameters. In addition to the expected outcome range, elevated values may arise from unfavorable combinations of uncertain inputs. This behavior likely results from the model’s pronounced non-linearity and underscores the importance of accurately characterizing input uncertainties to reduce uncertainty in the solutions.

Beyond the methodological contributions, the presented framework has potential implications for the understanding of HCC progression. By identifying parameters and regions that strongly influence blood flow and tumor growth, the approach may support a more targeted investigation of critical physiological mechanisms. In particular, the ability to quantify uncertainty in model predictions can contribute to a more reliable interpretation of simulation-based insights, which is essential for future patient-specific modeling and treatment planning.

In summary, the proposed framework successfully transforms a deterministic FEM simulation into an interpretable and efficient uncertainty-aware model. It enables the identification of key parameters and regions, supports probabilistic analysis under realistic physiological variability, and provides valuable information on model limitations.

Future work will focus on experimental calibration and refinement of the material formulation to better represent the mechano-biological interaction between stiffness, metabolism, and tumor growth. With regard to surrogate modeling, the extension of the GEK model for multivariate responses is an important aspect. On the one hand, this allows spatial dependencies of the underlying FEM model to be taken into account and, on the other hand, enables the emulation of the entire lobule.

## Supplementary information

Below is the link to the electronic supplementary material.Supplementary file 1 (zip 19 KB)

## Figures and Tables

**Fig. 10:**
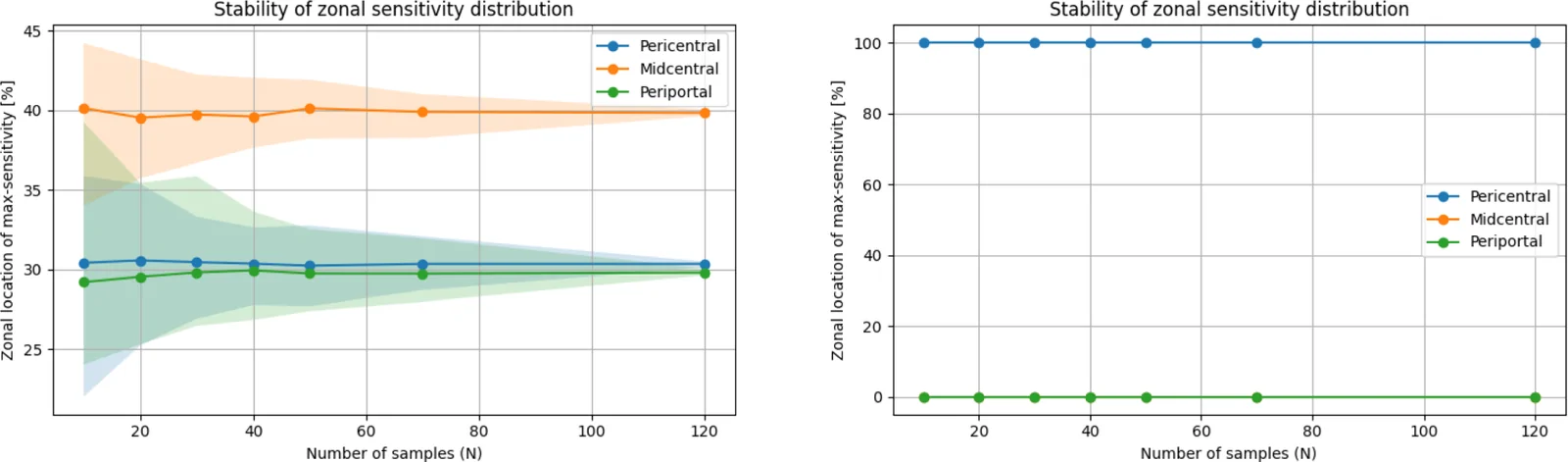
Stability of zonal sensitivity vs sample size for uncertain Young’s modulus; (left) for tumor volume fraction and (right) for blood velocity

**Fig. 11:**
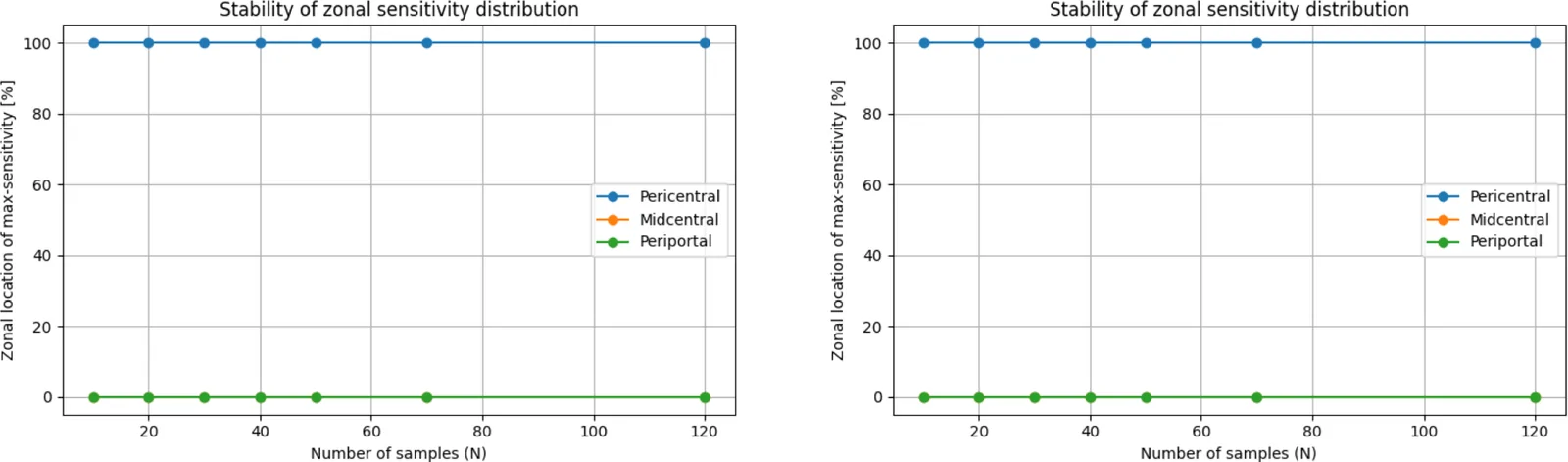
Stability of zonal sensitivity vs sample size for uncertain Darcy permeability; (left) for tumor volume fraction and (right) for blood velocity

**Fig. 12:**
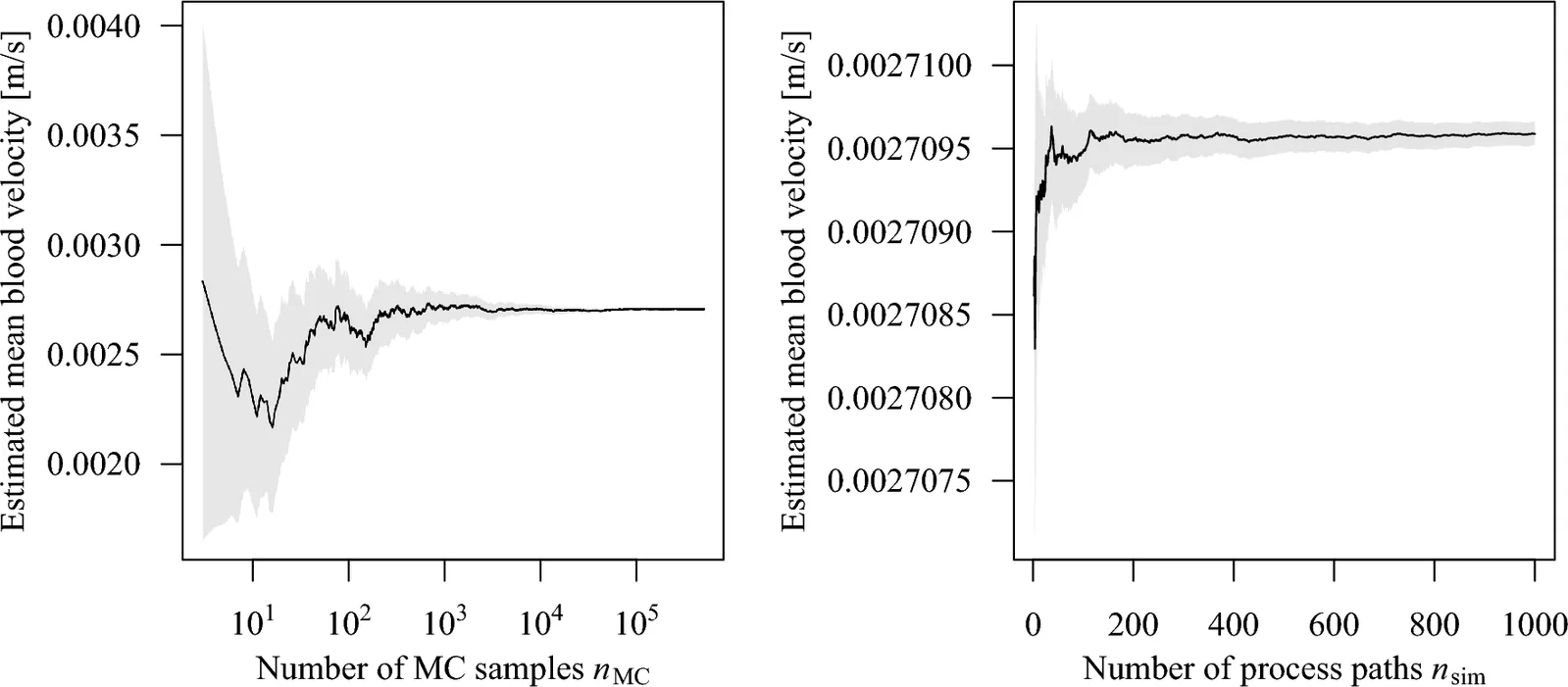
Monte Carlo (MC) convergence of the estimated mean blood velocity and approximated 95% confidence bounds with increasing MC samples (left) and process paths (right)

**Fig. 13:**
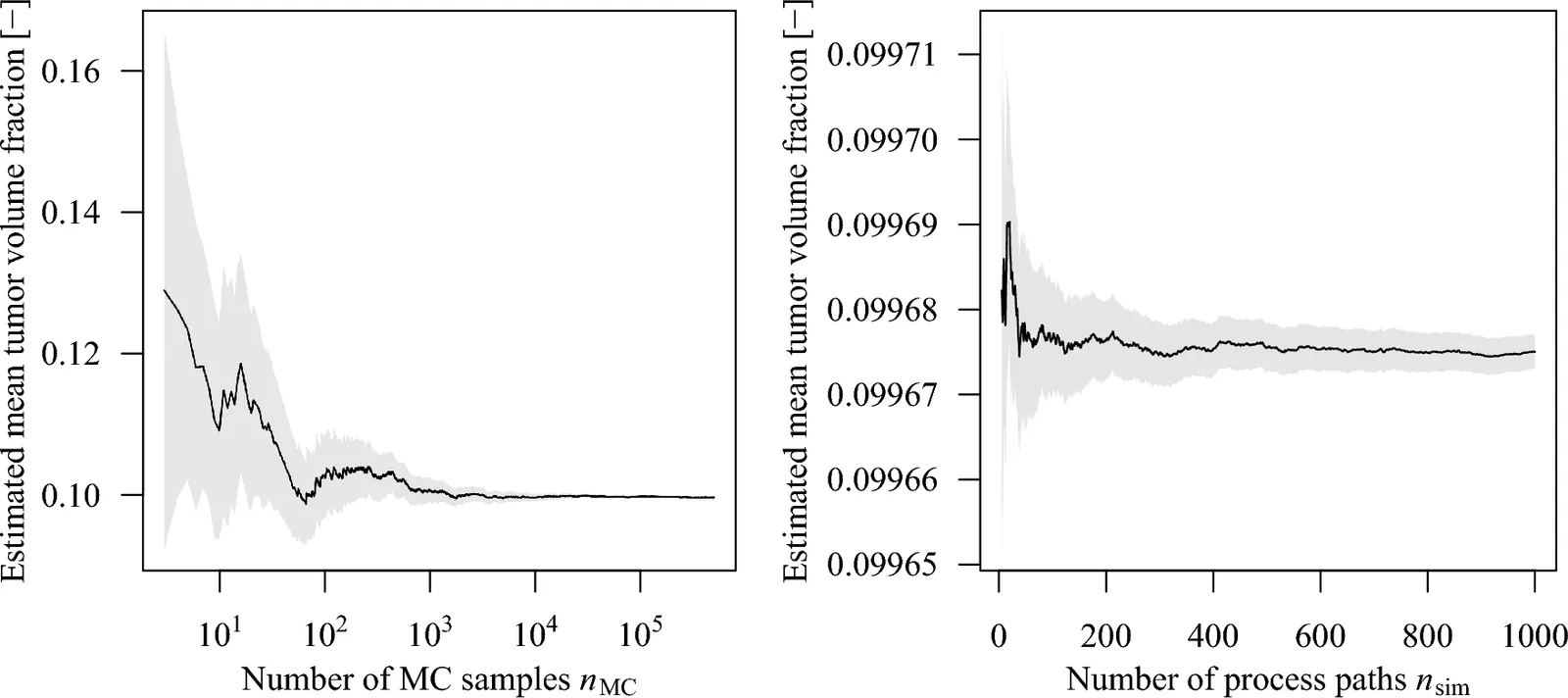
Monte Carlo (MC) convergence of the estimated mean tumor volume fraction and approximated 95% confidence bounds with increasing MC samples (left) and process paths (right)

## Data Availability

For reproducibility of the results, the training data and the associated program code for constructing and evaluation the surrogate models, as well as the subsequent uncertainty analyses are available in the supplementary material.

## Additional results

### Robustness of local sensitivity analysis

To further assess the robustness of the sensitivity analysis with respect to the number of simulations, an additional stability analysis was performed. Two independent uniformly sampled simulation sets (50 and 70 samples) were pooled and evaluated by repeated random subsampling for increasing sample sizes.

The analysis was carried out exemplarily for the Young’s modulus of liver tissue and the Darcy permeability, as these parameters were identified as representative and influential in the local sensitivity analysis.

The results show that the zonal sensitivity distribution for the tumor volume fraction, see Fig. 10 (left), exhibits some variability for small sample sizes, but stabilizes as the number of samples increases. In contrast, for blood velocity, see Fig. 10 (right), the location of maximum sensitivity remains consistently pericentral across all subsamples and sample sizes. A similar behavior is observed for uncertain Darcy permeability, leading to a zonal sensitivity distribution of the tumor volume fraction and blood velocity, see Fig. 11. Overall, the results confirm that the identified dominant regions and qualitative trends are robust with respect to the number of samples and the specific sampling realization.

### Convergence of MC simulations

The convergence of the MC simulations is examined based on the estimated expected values of the blood velocity and the tumor volume fraction for a successively increasing number of MC samples $n_{\mathrm{MC}}$ and process paths $n_{\mathrm{sim}}$. In order to reduce the computational effort, the MC samples are organized in batches of size $n_{\mathrm{batch}} = 4000$. For each batch $n_{\mathrm{sim}} = 1000$ conditional process paths are generated. The generated samples from the individual runs are then combined. Next, the cumulative means of the blood velocity and the tumor volume fraction are calculated separately for an increasing number of MC samples by averaging over the number of process paths, and vice versa.

Figure 12 displays the convergence of the estimated mean blood velocity for an increasing number of MC samples and generated process paths. The results show that the estimated mean values stabilize as both the number of MC samples and the number of process paths increase, while the confidence intervals narrow. Similar results are obtained for the tumor volume fraction, see Fig. 13.

For both blood velocity and tumor volume fraction, the estimated means converge from a sample size of $n_{\mathrm{MC}} = 10^4$ or larger, with narrow confidence intervals. Furthermore, the estimated means stabilize for an increasing number of process paths. This confirms that a total of $n_{\mathrm{MC}} = 5 \times 10^5$ MC samples and $n_{\mathrm{sim}} = 1000$ generated process paths are sufficient for the MC simulations.
